# Co-delivery of streptomycin and hydroxychloroquine by labeled solid lipid nanoparticles to treat brucellosis: an animal study

**DOI:** 10.1038/s41598-023-41150-0

**Published:** 2023-08-28

**Authors:** Narjes Morovati Moez, Seyed Mostafa Hosseini, Fereshte kalhori, Leili Shokoohizadeh, Mohammad Reza Arabestani

**Affiliations:** 1 Infectious Disease Research Center, Hamadan University of Medical Sciences, Hamadan, Iran; 2 Department of Microbiology, Faculty of Medicine, School of Medicine, Hamadan University of Medical Sciences, Hamadan, Iran; 3 Department of Anatomical Sciences, Faculty of Medicine, Hamadan University of Medical Sciences, Hamadan, Iran

**Keywords:** Biotechnology, Drug discovery, Genetics, Microbiology

## Abstract

Can brucellosis-related biochemical and immunological parameters be used as diagnostic and treatment indicators? The goal of this project was to look at biochemical parameters, trace elements, and inflammatory factors in the acute and chronic stages of brucellosis after treatment with streptomycin and hydroxychloroquine-loaded solid lipid nanoparticles (STR-HCQ-SLN). The double emulsion method was used for the synthesis of nanoparticles. Serum levels of trace elements, vitamin D, CRP, and biochemical parameters were measured in rats involved in brucellosis. The therapeutic effect of STR-HCQ-SLN was compared with that of free drugs. In both healthy and infected rats, serum concentrations of copper, zinc, iron, magnesium, potassium, and biochemical parameters of the liver were significantly different. By altering the serum levels of the aforementioned factors, treatment with STR-HCQ-SLN had a positive therapeutic effect on chronic brucellosis. Vitamin D levels declined (46.4%) and CRP levels rose (from 7.5 mg to less than 1 mg) throughout the acute and chronic stages of brucellosis. This study showed that by comparing the biochemical parameters and the levels of trace elements in the serum of healthy and diseased mice in the acute and chronic stages of brucellosis, it is possible to get help from other routine methods for diagnosis.

## Introduction

Brucellosis is a zoonotic disease that commonly affects humans and animals and is caused by various species of the genus *Brucella*. This disease can cause non-specific clinical symptoms, numerous side effects, and damage to various organs^1,2^. The four species of *Brucella* that have the potential to infect humans are *Brucella abortus*, *Brucella melitensis, Brucella suis*, and *Brucella canis*. *Brucella*, which live inside macrophages and are protected from the extracellular environment, are also able to become resistant to in vivo antibiotics that were only effective under in vitro conditions previously^3,4^. Cows, sheep, goats, and other small ruminants can excrete bacteria in their milk and secretions, causing infection in humans and other animals. One of the main ways of transmitting the disease to humans is through contact with infected animals and the consumption of unpasteurized dairy products. Even with great progress in brucellosis control in developed countries, this infection is still an important disease in the Middle East (ME)^5,6^.

Following *Brucella*'s entry into macrophages and preventing apoptosis, the ability of bacteria to adapt to new environmental conditions, bacterial growth in macrophage cells, the absence of host self-protection, and subsequent treatment failure make eradication of bacteria difficult, and thus re-infection occurs^7,8^. Within the first six months of treatment, nearly 30% of patients experience a milder disease recurrence than in the earlier stages. To this end, new approaches are needed to reduce disease recurrence^9^.

Utilizing nanocarriers is one promising strategy. The precise formulation of nanocarriers increases their stability and has the potential to accelerate their dissolution, adequate biological level, therapeutic effect, and bioavailability^10–12^. As a result, drug delivery systems are regarded as a promising method for overcoming the limitations of conventional treatment^13^.

Trace elements are essential for the body's nutrition, biochemistry, and enzyme structure. Determining the function of these elements during puberty, activity, and defense could be a promising tool in molecular biology and genetics^12^. The cells of the immune system need trace elements in sufficient quantities for proper structure and functioning^14,15^. For example, copper is essential for cytochrome C oxidase in the mitochondrial electron transport chain, and iron is required for succinate dehydrogenase (SDH) and cytochrome A, B, C, and NADH functions^16^. Improvement the activity of enzymes that enhance cell defense function requires trace elements^17^. Additionally, more than 300 enzymes involved in the biosynthesis of sugars, proteins, and fats require magnesium, which is necessary for their stability and proper function^12^.

Vitamin D is another important trace element because it helps maintain calcium homeostasis, which is critical for both the innate and acquired immune systems^18^. According to studies, a healthy level of vitamin D in the host's serum increases the strength of the immune system against infections. Vitamin D levels in the blood have been found to be correlated with infection risk. Additionally, vitamin D aids macrophage function and this vitamin is required for the INF1 antimicrobial activity in human macrophages^19^.

Brucellosis has destructive effects on human body organs such as the liver, spleen, joints, etc. As a result, the amount of enzymes and proteins in the body also changes. For example, the C-reactive protein (CRP) level in the serum typically falls below 1 mg/L in healthy adults; however, the CRP level rapidly rises in the majority of infectious diseases. Brucellosis causes destruction of liver enzymes such as alanine transaminase, total bilirubin, alkaline phosphatase, and aspartate aminotransferase.

Solid lipid nanoparticles (SLN) have been concentrated as a carrier framework for many applications. Various molecules, peptides, and drugs are successfully loaded into SLNs. The drug delivery system regulates the drug release and also improves the chemical stability of the drug^20^. In this regard, the aim of this in vivo experiment was to determine the therapeutic effect of SLN containing streptomycin (STR) and hydroxychloroquine (HCQ) (STR-HCQ-SLN) on acute and chronic phases of brucellosis and its effect on serum levels of C-reactive protein, vitamin D, renal function parameters and liver enzymes.

## Materials and methods

### STR-HCQ-SLN synthesis

The double emulsion/melt dispersion technique was used to prepare nanoparticles^10^. To achieve optimal formulation, different amounts of two types of lipids, water-soluble and lipid-soluble surfactants, and drugs were evaluated***.*** Briefly, palm oil or stearic acid is first heated to 70 °C using a double boiler (a type of hot water bath). The lecithin/poloxamer and drugs (streptomycin and HCQ) were then added to the molten oil and mixed with a magnetic stirrer. The mixture was homogenized after the addition of heated distilled water and sonication at 45 °C for 60 s. In the second step, the prepared initial emulsion was mixed with pre-heated Tween-80, which was homogenized with an ultrasonic device for one minute at 45 °C. In this step, the final prepared emulsion was slowly added to the solution of distilled water and quantum dot cadmium telluride at 4 °C, which was placed on a magnetic stirrer, to stabilize the synthesized nanoparticles. The same process was performed to prepare free SLN. Finally, the synthesized nanoparticles were washed three times with distilled water in a high-speed centrifuge (35,000 rpm for 20 min).

### Nanoparticle properties

After the synthesis of STR-HCQ-SLN, its characteristics, such as average size, zeta potential, and polydispersity index, were evaluated. The maximum wavelength (λmax) of STR and HCQ was also assessed. Then, a standard curve was drawn using a spectrophotometer at the λmax of each drug. Direct (spectrometer) and indirect (high-performance liquid chromatography (HPLC)) methods were used to determine the amount of encapsulation and loading of drugs in SLN. The thermal index of the nanoparticle and its components was examined using a differential scanning calorimetry (DSC) (METTLER TOLEDO-DSC 1). For spectroscopic examination of the samples, the lyophilized powder of synthesized nanoparticles and nanoparticle components was mixed with a certain amount of potassium bromide (kb) at the same time and turned into compressed disks by a hydraulic compressor. The discs were then recorded using fourier-transform infrared spectroscopy (FTIR) (PerkinElmer Spectrum 400, America) in the mid-IR (400–4000 cm) range of the infrared light path. The dialysis bag method was used to evaluate the drug release rate from the synthetic nanoparticle substrate (14,000–12,000 Dalton molecular weight and 2.5 nm pore size).

Both the short-term (a week of review in terms of appearance and one month in terms of physicochemical properties and appearance) and long-term (from aspects of physicochemical properties and appearance, six to twelve months) aspects of nanoparticle stability were examined. J774-A.1 mouse macrophage cell line was used to evaluate the cytotoxicity of the synthesized nanoparticles according to the instructions of the kit (Kiasyst Kit/Iran). The morphology of the nanoparticles was observed using a Field emission scanning electron microscopy (FE-SEM)^10,21^.

### Animal testing (in vivo condition)

Male Wistar rats weighing 250 ± 25 g and ranging in age from 6 to 8 weeks were obtained from Hamadan University of Medical Sciences' animal house. *B. melitensis* strain m16 at a concentration of 1.5 × 10^6^ CFU/ml was inoculated into mice by intraperitoneal injection (IP). After 10 days (the acute infection phase) and 6 weeks (the chronic infection phase), mice were divided into 8 groups.

Five rats from each group were studied during the acute phase of infection: (1) a positive control (the untreated infected group), (2) a negative control (the healthy rats), (3) treated with free STR (2.5 mg/kg), (4) treated with free HCQ (2.6 mg/kg), (5) treated with free STR and HCQ, (6) treated with STR-SLN (2.5 mg/kg), (7) treated with HCQ-SLN, and (8) treated with STR-HCQ-SLN eleven, thirteen, and fifteen days after infection.

Moreover, ten rats from each group were evaluated during the chronic phase of infection: (1) a positive control (the untreated infected group), (2) a negative control (the healthy rats), (3) treated with free STR (2.5 mg/kg), (4) treated with free HCQ (2.6 mg/kg), (5) treated with free STR and HCQ, (6) treated with STR-SLN (2.5 mg/kg), (7) treated with HCQ-SLN, and (8) treated with STR-HCQ-SLN for 10 days (once daily). One day after the last parenteral dose, 87/13 mg/kg ketamine and xylazine were used to anesthetize rats. Blood from the hearts of rats was taken for further study^20^. Animal studies were performed in accordance with the ARRIVE guidelines. The ethics committee of Hamadan University of Medical Sciences gave its approval for this study, in accordance with the US National Institutes of Health guidelines. Considering the pathogenic nature of Brucella, all safety protocols, including Biosafety Level 3 (BSL-3) and the use of appropriate Personal protective equipment (PPE), were considered.

### Measurement of trace elements

Serum levels of trace elements (zinc, copper, iron, calcium, phosphorus, and magnesium) were determined photometrically using an Audit Kit (Ireland) and an AutoAnalyzer (Hitachi 902, Japan). Serum sodium and potassium levels were then measured using Convergys® ISE (Germany)^12^.

### Serum levels of biochemical parameters

Serum levels of aspartate aminotransferase (AST), alanine transaminase (ALT), alkaline phosphatase (ALP), creatinine, total bilirubin (TBil), and urea were determined using the Audit kit (Ireland) and AutoAnalyzer (Hitachi 902, Japan) with the photometry method^12^.

### Measurement of CRP and vitamin D

Serum CRP levels in rats were checked using the NycoCard™ CRP kit (Norway). This kit is an immunochemical assay for the quantitative determination of CRP in whole blood, serum and plasma. CRP measurement provides information for the diagnosis and evaluation of infections, inflammatory disorders, and related diseases. To measure the vitamin D levels in different study groups, the Elisa kit (Arka Fanazma-Iran) was used according to the kit instructions^18^.

### Statistical analysis

The mean and standard deviation of all studied serum parameters were calculated at the acute and chronic stages of the disease. The groups were compared using Duncan's multiple range and Analysis of variance (ANOVA) tests because there were multiple groups in the two stages. The relationship between the different parameters was obtained by Pearson's correlation analysis and *P* value ≤ 0.05 were considered significant.

### Ethics statement

The study was conducted under the ethics approval code IR.UMSHA.REC.1401.916.

## Results

### Nanoparticles characterization, encapsulation and drug loading rate

**Figure 1:**
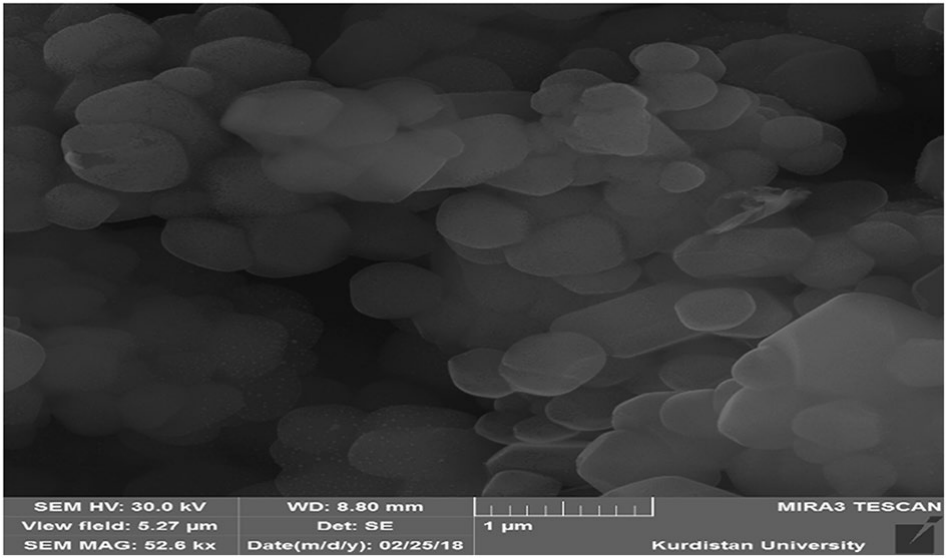
FE-SEM image of optimum formulation.

The average size and the optimal polydispersity index (PDI Index) of the optimal nanoparticles (F4) were 312.5 ± 26 and 0.433 ± 0.09 nm, respectively. The optimal nanoparticles (F4) encapsulated STR and HCQ at rates of 94.2 and 85%, respectively, with a zeta potential of − 15.2 mV. The mean drug loading rate in the different formulations was 13.07%. The loaded content in the F4 formulation was 16.6% the optimal nanoparticles, according to the findings of FE-SEM, had a morphologically spherical shape, a smooth surface, and uniform dispersion. (Table 1, Fig. 1).

**Table 1:** Details of the amount of materials used and their results in the synthesized formulation.

Formulation	Streptomycin (Mg)	Hydroxychloroquine (Mg)	Palm oil (Mg)	Size (nm)	PDI	Zeta potential (mV)	Average drugs encapsulation (%)	Average drugs load (%)
F1	60	60	500	460.2	0.288	− 15.5	91.1	15.3
F2	30	30	300	380.2	0.322	− 11.6	93.6	11.1
F3	120	60	1200	389.4	0.422	− 14.0	89.3	12.8
F4	**120**	**120**	**1200**	**312.5**	**0.433**	**− 15.2**	**89.6**	**16.6**
F5	60	120	1000	394.9	0.380	− 17.5	91.7	11.1
F6	120	120	2400	460.7	0.499	− 15.7	82.8	11.1
F7	240	120	2400	604.4	0.599	− 12.1	90.7	12.9
F8	120	240	1200	450.3	0.256	− 15.9	86.7	9.7

Significant values are in [bold].

### Toxicity

**Figure 2:**
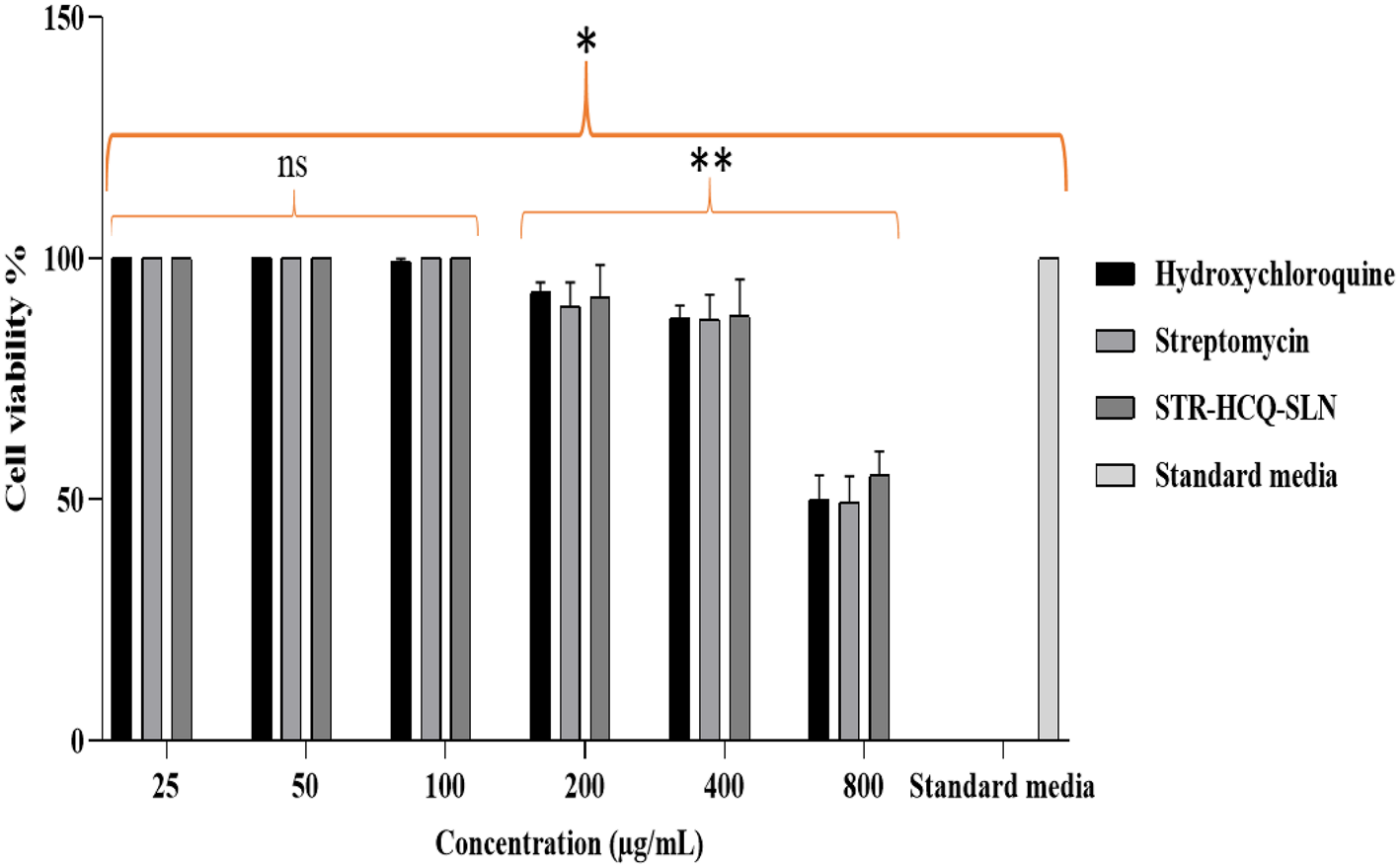
The effect of free drugs and nanoparticles on J774A.1 cell. *P value < 0.05, **P value < 0.001).

Toxicity test outcomes showed that the free drug and synthesized nanoparticles at a concentration of 100 mg/mL had no toxic effects. Furthermore, the effects of nanoparticle and free drug on the cells were not statistically different (*P* > 0.05) (Fig. 2). Notably, according to the minimum inhibitory concentration (MIC) results, the antibacterial doses in in vivo and in vitro conditions were lower than the doses used in the toxic test.

### Parameters of liver and kidney function

The serum level of liver enzymes was significantly different between the positive control rat group and the healthy rat group. The positive control group had a 65% increase in the serum level of AST compared to healthy rats. Treatment with STR-HCQ-SLN significantly decreased the AST level (*P* < 0.001). Furthermore, rats treated with free STR had lower levels of AST, but this decrease was statistically insignificant (*P* > 0.05) in comparison to positive control. The serum levels of TBil and ALP in the groups treated with STR-HCQ-SLN and STR-SLN showed a significant decrease in comparison to the positive control group. The serum levels of ALT did not change much in the various groups that were examined in this study. According to Table 2, there was no statistically significant difference (*P* < 0.05) between serum levels of urea and creatinine in different groups.

**Table2:** Biochemical and kidney function parameters in different treatment groups.

Group/parameter	Stage of disease	AST (U/L)	ALT (U/L)	ALP (U/L)	TBil (mg/dL)	Urea (mg/dL)	Creatinine (mg/dL)
Positive control	Acute	173 ± 4.01	84 ± 5.01	1150 ± 35. 2	0.45 ± 0.01	54.3 ± 1.5	0.41 ± 0.04
	Chronic	161 ± 5.03	75 ± 2.02	930 ± 11. 3	0.41 ± 0.02	44.3 ± 0.5	0.52 ± 0.03
Free STR	Acute	132 ± 5.02	81 ± 7.20	870 ± 9.02	0.23 ± 0.009	51.1 ± 1.7	0.42 ± 0.05
	Chronic	141 ± 7.08	68 ± 4.04	810 ± 31. 60	0.22 ± 0.01	49.3 ± 2.1	0.48 ± 0.03
Free HCQ	Acute	163 ± 2.4	84 ± 3.9	1130 ± 302.6	0.41 ± 0.02	44.8 ± 2.6	0.47 ± 0.02
	Chronic	152 ± 5.01	74 ± 67.01	850 ± 16. 32	0.39 ± 0.01	43.4 ± 1.9	0.53 ± 0.02
Free STR & HCQ	Acute	121 ± 6.8	82 ± 7.80	810 ± 11.8	0.26 ± 0.01	48.1 ± 3.1	0.49 ± 0.05
	Chronic	143 ± 2.90	67 ± 3.23	815 ± 35. 46	0.22 ± 0.01	45.3 ± 2.5	0.49 ± 0.06
STR-SLN	Acute	119 ± 7.06*	77 ± 5.02	610 ± 206.1*	0.17 ± 0.00*	58.3 ± 2.8	0.52 ± 0.03
	Chronic	95 ± 1.01*	68 ± 4.11	516 ± 13. 5*	0.15 ± 0.02*	48.1 ± 2.1	0.53 ± 0.04
HCQ- SLN	Acute	157 ± 4.6	81 ± 8.0	980 ± 20.3	0.39 ± 0.02	49.6 ± 3.9	0.47 ± 0.02
	Chronic	126 ± 5.22	72 ± 7.01	842 ± 20. 75	0.33 ± 0.03	49.3 ± 2.5	0.45 ± 0.01
STR-SLN-HCQ	Acute	115 ± 2.2**	76 ± 4.1	523 ± 30.2**	0.13 ± 0.01**	51.5 ± 2.6	0.48 ± 0.03
	Chronic	94.6 ± 3.7**	64 ± 3.01	416 ± 11. 1**	0.10 ± 0.02**	40.2 ± 1.7	0.48 ± 0.12
Healthy rat	Acute	105 ± 2.0**	75 ± 3.8	423 ± 10.5**	0. 09 ± 0.02**	55.7 ± 1.8	0.42 ± 0.03
	Chronic	90.3 ± 4.04**	62 ± 4.12	400 ± 18.61**	0.12 ± 0.03**	45.3 ± 2.31	0.43 ± 0.11

*AST* aspartate transaminase, *ALT* alanine transaminase, *ALP* alkaline phosphatase, *TBil* total bilirubin, *STR* streptomycin, *HCQ* hydroxychloroquine, *SLN* solid lipid nanoparticles.

Results are represented as mean ± SD.

*p < 0.05 and ** *p* < 0.001, significantly compared to the positive control.

### Determination serum levels of K, Na, Ca and Pho (trace element)

**Figure 3:**
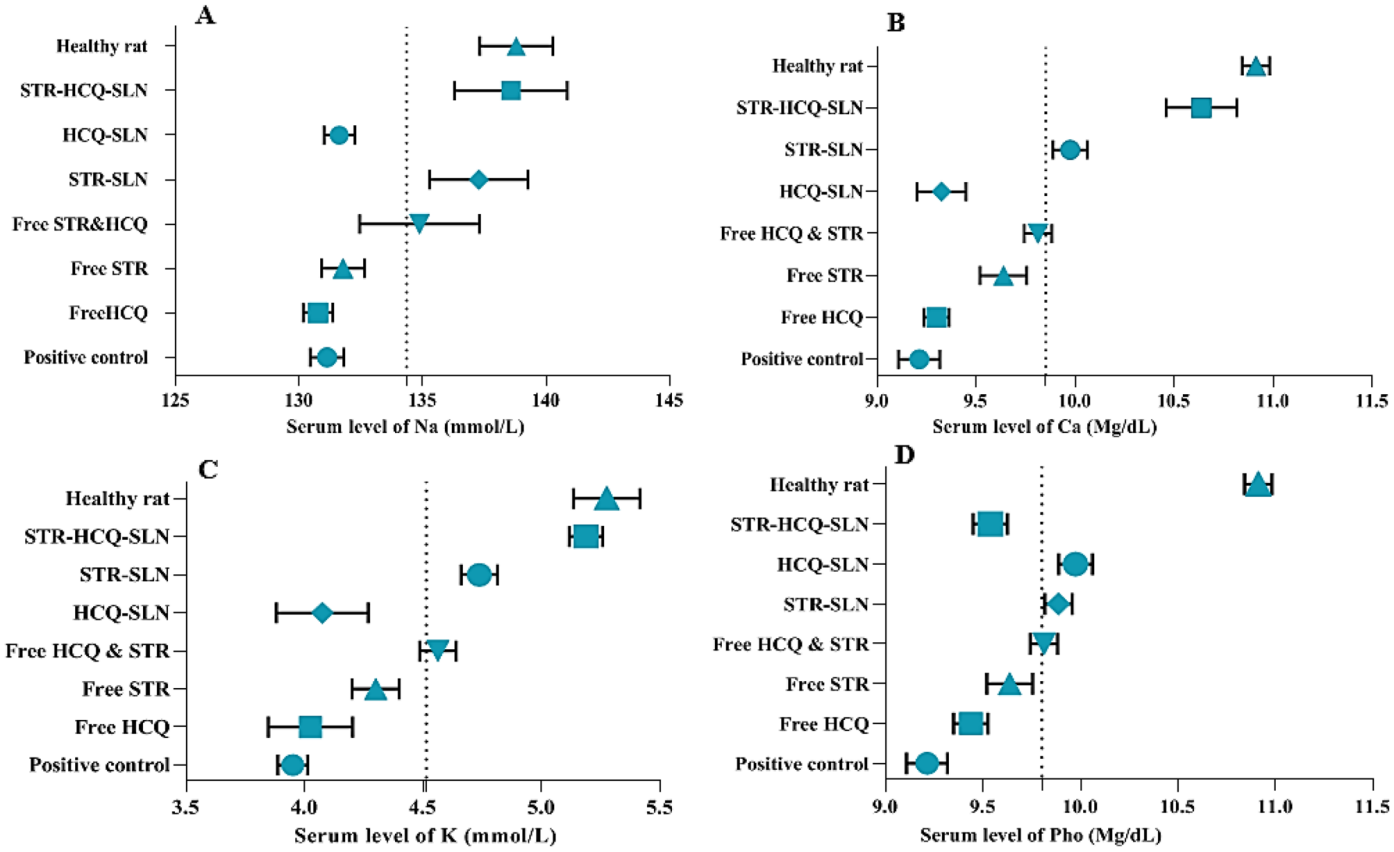
Levels of trace elements in the serum; in the acute and chronic stages of brucellosis. Sodium (**A**), calcium (**B**), potassium (**C**), and phosphorus (**D**).

The data indicated that the healthy rats had more sodium levels than the positive control (*P* < 0.05). Sodium levels in the STR-HCQ-SLN-treated rats were nearly identical to those in the Healthy rat group. Furthermore, serum calcium levels in STR-HCQ-SLN-treated rats were significantly closer to those in healthy rats. Serum potassium level was the same as sodium level. The STR-HCQ-SLN-treated group experienced the highest level of recovery, and the drug was able to restore healthy rats' serum potassium levels to their normal range. Serum levels of phosphorus did not differ significantly between healthy and control groups, according to the findings. Furthermore, no remarkable differences were observed in the serum concentrations of the trace elements mentioned in the different groups and in the chronic and acute phases (Fig. 3).

### Serum levels of Cu, Zn, Fe and Mg

**Figure 4:**
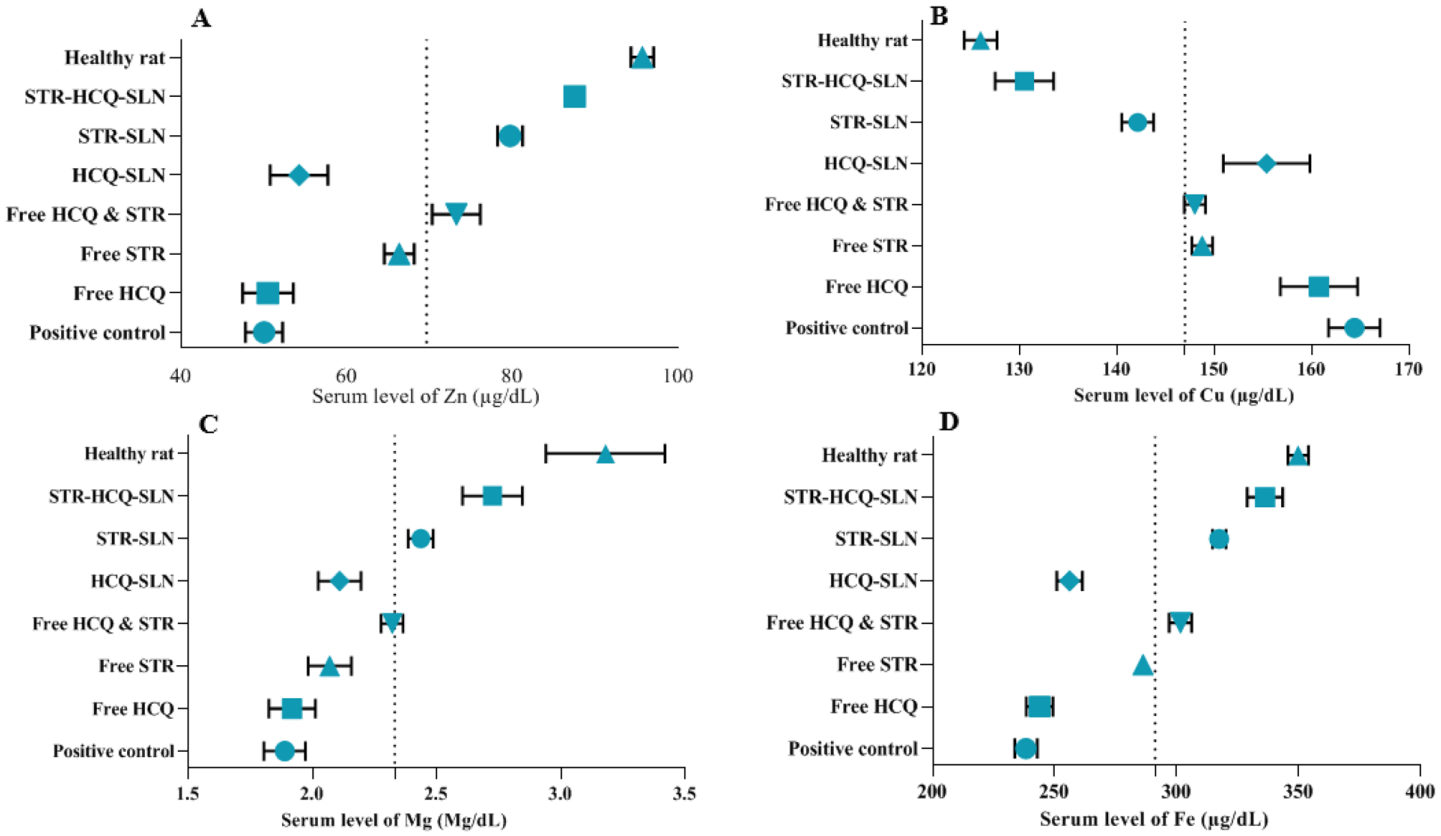
Trace element concentration in the serum during the acute and chronic stages of brucellosis. Zn (**A**), Cu (**B**), Mg (**C**), and Fe (**D**) concentrations.

Figure 4 shows that the positive control group's serum zinc (Zn) level is significantly lower than those in healthy rats (*P* < 0.05). The serum level of zinc was notably raised in STR-HCQ-SLN treated rats in acute and chronic brucellosis (*P* < 0.05). In both the acute and chronic phases, the infected groups had an increased in the amount of copper (Cu) in comparison to the healthy rats. In contrast, serum iron (Fe) and magnesium (Mg) levels were significantly decreased during the acute and chronic stages of brucellosis. The group treated in this study with free drugs and synthesized nanoparticles generally normalized the level of trace elements. In conclusion, to compare the groups, STR-SLN and STR-HCQ-SLN had the greatest impact on the recovery of rats.

### Vitamin D and CRP levels in the serum

**Figure 5:**
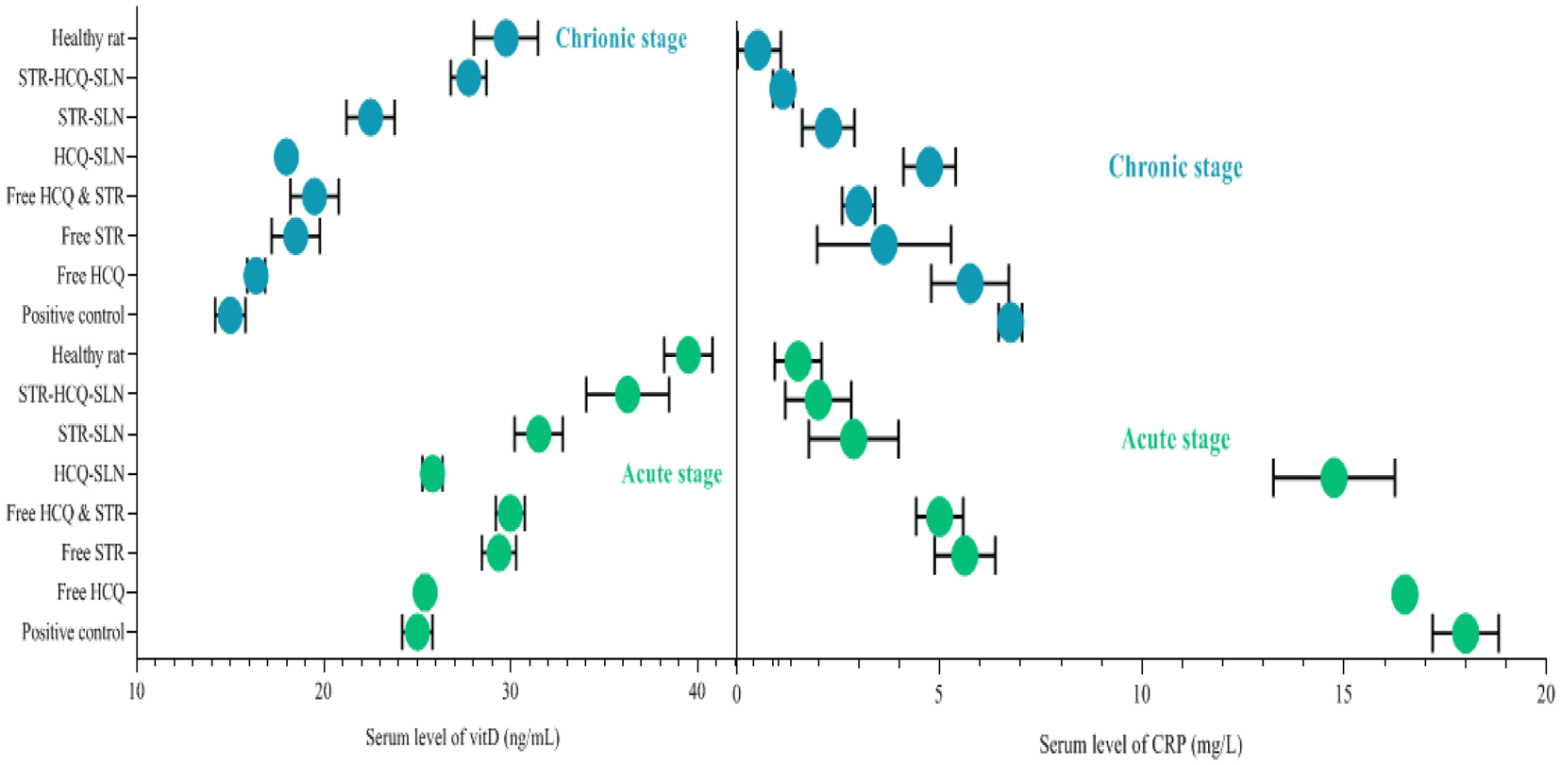
Serum level of Vitamin D and CRP in the acute and chronic stage of brucellosis.

Between the healthy rats and the positive control group, the serum vitamin D level had remarkably decrement (*P* < 0.05). Furthermore, rats showed significantly lower serum vitamin D levels during the chronic phase compared to the acute phase. Finally, the use of STR-HCQ-SLN drug increased vitamin D in infected rats. CRP increased in both disease stages in infected rats (*P* = 0.001). Treatment of mice with STR-HCQ-SLN, STR-SLN and free STR reduced CRP levels during the inflammatory phase of infected rats (Fig. 5).

## Discussion

Trace elements are known to be important building blocks of the body and regulators of the immune system, which can regulate the body's sensitivity directly or indirectly to viral and bacterial infections. For example, all immune cells, including B cells, T cells, neutrophils, and macrophages, have vitamin D receptors on their cell surfaces. When macrophages are exposed to LPS in bacterial cells, genes that produce vitamin D begin to be expressed. The synthesis of cathelicidin, an antimicrobial peptide, is initiated by increasing vitamin D production. The produced cathelicidin assists the immune system of the host in eliminating the foreign pathogen. In addition, many enzymes and hormones require trace elements for their function. Studies have shown that small changes in serum levels of trace elements can adversely affect the host's immune cells. The immune system is unable to fight microorganisms as a result of the body's weakening of the body's trace elements against infectious agents caused by fluctuations in serum concentration.

The liver is one of the host body's crucial organs that can be affected by brucellosis. Brucella bacterial cells are phagocytized by hepatic macrophages and the bacteria's intracellular activity results in the death of liver cells and the release of liver enzymes. In addition, Brucella's intracellular growth and the acidic conditions in the phagosomes of macrophages have enabled the infectious agent to evade the effects of extracellular antibiotics and remain concealed within the macrophages for months. On the other hand, nano-drug delivery systems help antibacterial agents penetrate macrophages longer by slowly and continuously releasing drugs specifically at target sites.

In this study, rats infected with *B. abortus* in different groups (acute and chronic stages of the disease) were treated with free drugs and nano-carrier drugs. Serum levels of minerals, liver and kidney enzymes, vitamin D and CRP were also measured.

In our study, a repeatable, cheap and simple method called double emulsion was utilized to plan nanoparticles, which requires natural solvents and a limited quantity of surfactant. Hydrophilic drugs like STR and HCQ can be encapsulated using double emulsion.

The prepared STR-HCQ-SLN nanoparticles have a diameter of (26 ± 312.5), which is a reasonable size for absorbing phagocytic cells. The study found that the size of nanoparticles decreases as the ultrasound probe time increases, which is consistent with the findings of Liu et al. [27]. Additionally, the present study demonstrated that drug loading increased the size of the STR-HCQ-SLN in comparison to the free drug. Also, after lyophilization, the size of the nanoparticles increased, which is consistent with the findings of the Chantaburanan et al. study^16^. Severino et al. used a high pressure homogenization method to create nanoparticles with a diameter of 439.5 nm [28]. When the PDI value approaches zero, the particles become more homogeneous. The factor that increases the stability of nano-drug as a treatment is high zeta potential. Nano-drug stability is influenced by zeta potential, a crucial physicochemical property (18). In the present study, the nanoparticles had an electrical charge of − 15.2 mV. This finding shows less toxicity and the high yield of encapsulation without using organic solvents.

In 2022, Elbehiry et al. investigated the effect of silver and gold nanoparticles on *Brucella mellitenensis* and *Brucella abortus* strains. Using the MIC method, they found that silver and gold can have an inhibitory effect on the growth of the mentioned bacterial strains. In the current study, the effectiveness of nanoparticles has been compared with the free drug, while Elbehiry et al. did not conduct this investigation and only nanoparticles were investigated. Another important point is that Elbehiry and his colleagues found out by examining the histopathology of gold and silver nanoparticles that the use of 2 mg of it had a toxic effect on the tissues of laboratory animals (rats) which is contrary to the findings of the present study^5^.

Different groups showed an increase in liver enzyme levels in both the acute and chronic stages of the disease, according to the study's findings. In comparison to healthy mice, whose serum level was 105 U/L, the AST level in the acute phase in this study was 173 U/L in the positive control group and 115 U/L in the STR-HCQ-SLN group. This article shows the good effect of STR-HCQ-SLN on increasing the serum level of AST to the normal range.

In the acute stage of the disease, the positive group's serum levels of AST, ALP, and TBil increased by 10.5, 271, and 346 percent, respectively, in comparison to the healthy group. This increase was also evident during the chronic phase of brucellosis (*P* < 0.05). Table 2 shows that the STR-SLN treated group had significantly decreased serum levels of trace elements compared to the positive control (*P* < 0.05), but the treatment effect of lowering liver enzymes and total bilirubin was highest in the group using STR-HCQ-SLN (*p* < 0.001). Similar to Bozukluhan and colleagues' findings, our results show an increase in AST and ALT of 42 and 61%, respectively, following infection [24].

Singa et al. infected cattle with strains of brucellosis. ALT and AST levels increased by 20 and 15%, respectively, in line with this study's findings [25]. In addition, liver enzyme levels have been found to rise in both the acute and chronic phases of brucellosis, as reported in other articles^10,16^. Summarizing the results this study's findings and those of other articles that are related to it, it can be concluded that measurement of liver enzyme levels may be an appropriate marker for diagnosis of brucellosis. The fact that enzyme levels decrease more rapidly in the chronic phase than in the acute phase indicates that brucellosis takes longer to treat. The findings indicate that HCQ can increase STR's efficiency and effectiveness against Brucella, thereby accelerating the healing process. Trace elements play an important role in the protecting the body against many infectious diseases [29].

In this study, the serum zinc levels in the acute and chronic phases of brucellosis decreased by approximately 90%, which increased after treatment with Free STR, Free STR-HCQ, STR-SLN, and STR-HCQ-SLN. In general, there was no discernible difference in trace element levels between the control group and brucellosis-infected rats treated with Free HCQ or HCQ-SLN. In fact, the interleukins released by leukocytes and phagocytes induce active transport of zinc from the blood to the liver, and as a result, the serum level of zinc (Zn) decreases in infectious diseases [30]. Additionally, the group that received STR-HCQ-SLN experienced the greatest elevation in serum sodium levels. Also, after infecting rats with Brucella, the serum potassium level decreased significantly. After several experimental tests, STR-HCQ-SLN showed the best therapeutic effect compared with the positive group. When compared to healthy groups, there were no significant changes in serum copper levels in both acute and chronic disease conditions (*P* > 0.05).

When compared to the group of healthy rats, our research revealed that in both the acute and chronic stages of the disease, the serum Fe level significantly decreased, while in the chronic stage, the serum Fe level significantly increased following treatment with STR-HCQ-SLN. Additionally, in the Bozukluhan et al. study, in accordance with the findings of this study, cows with brucellosis had a 50% lower Fe serum level than healthy groups [24]. It should be noted that low Fe levels cause liver hurt and dysfunction, as well as increased Fe uptake by bacteria [29]. There was no remarkable difference in serum levels of Ca and Ph in different groups in both acute and chronic stages.

This study found that healthy rats had higher serum vitamin D levels than rats in the acute and chronic stages of the disease. A comprehensive investigation into the effects of rifampin-carrying nanoparticles on vitamin D serum levels in Brucellosis was carried out in 2019 by Arabestani and colleagues. The results showed that serum vitamin D levels decreased sharply in rats infected with Brucella, then increased after treatment^16^. Similar results were obtained from the study by Beltran et al., showing that serum vitamin D levels were 9.6 ng lower in patients with brucellosis than in healthy individuals. [26]. the serum vitamin D level of tuberculosis patients and healthy subjects were measured and compared in another study that is consistent with ours. According to the findings, the patient group's serum vitamin D level was 15 ng/dL, significantly lower than the range of 17 ng/dL for healthy people in the control group [27]. Another study by Atai et al. in 2016 evaluated serum vitamin D levels in people with hepatitis C. In this research, the serum vitamin D level in healthy individuals was 29.6 ng/dL, which is higher than vitamin D levels in HCV positive patients with a range of 26.23 ng/dL [28].

Some patients with chronic liver cirrhosis do not have adequate serum vitamin D levels [26]. In our study, a group of patients showed a significant increase in serum vitamin D levels after treatment with STR-HCQ-SLN during the acute and chronic phases of the disease. STR-HCQ-SLN can provide superior therapeutic efficacy. When compared to the healthy mice in the control group, the level of CRP in the positive control group increased during both the acute and chronic stages of the current study. Serum CRP level was evaluated before and after brucellosis treatment [31]. According to the findings of Demirdag et al.’s investigation of the serum level of CRP in acute brucellosis, the serum level significantly decreases from 52.4 mg/liter during the acute phase of the disease to 11 mg/liter following treatment [32].

## Conclusion

In both the acute and chronic stages of brucellosis, the present study shows that the use of STR nanoparticles and HCQ simultaneously can be more effective as a treatment. By measuring and comparing the serum levels of biochemical parameters and trace elements in healthy and diseased mice, it is suggested that these parameters can be used together with other diagnostic methods such as culture and serological tests to diagnose and monitor the treatment of brucellosis.

## Data Availability

The original contributions presented in the study are included in the article/Supplementary Material; further inquiries can be directed to the corresponding author.

## Abbreviations

SDH: Succinate dehydrogenase
CRP: C-reactive protein
T4SSs: IV secretion systems
SLN: Solid lipid nanoparticle
STR: Streptomycin
HCQ: Hydroxychloroquine
HPLC: High performance liquid chromatography
FTIR: Fourier transform infrared spectroscopy
Fe-SEM: Field emission scanning electronic microscope
DLS: Dynamic light scattering
NaBH4: Sodium borohydride
DDW: Double distilled water
TGA: Thioglycolic acid
CdTe-QDs: Cadmium telluride quantum dot
PDI: Polydispersity index
DSC: Differential scanning calorimetry
AST: Aspartate aminotransferase
ALT: Alanine transaminase
TBil: Total bilirubin
